# Supplementary material for: Characteristics and Comparative Analysis of Seven Complete Plastomes of Trichoglottis s.l. (Aeridinae, Orchidaceae)
Source: Int J Mol Sci. 2023 Sep 26;24(19):14544. doi: 10.3390/ijms241914544 (PMC10572978; doi:10.3390/ijms241914544)
Supplement: Supplementary file 1 [file ijms-24-14544-s001.zip › Supplementary Figure.pdf]

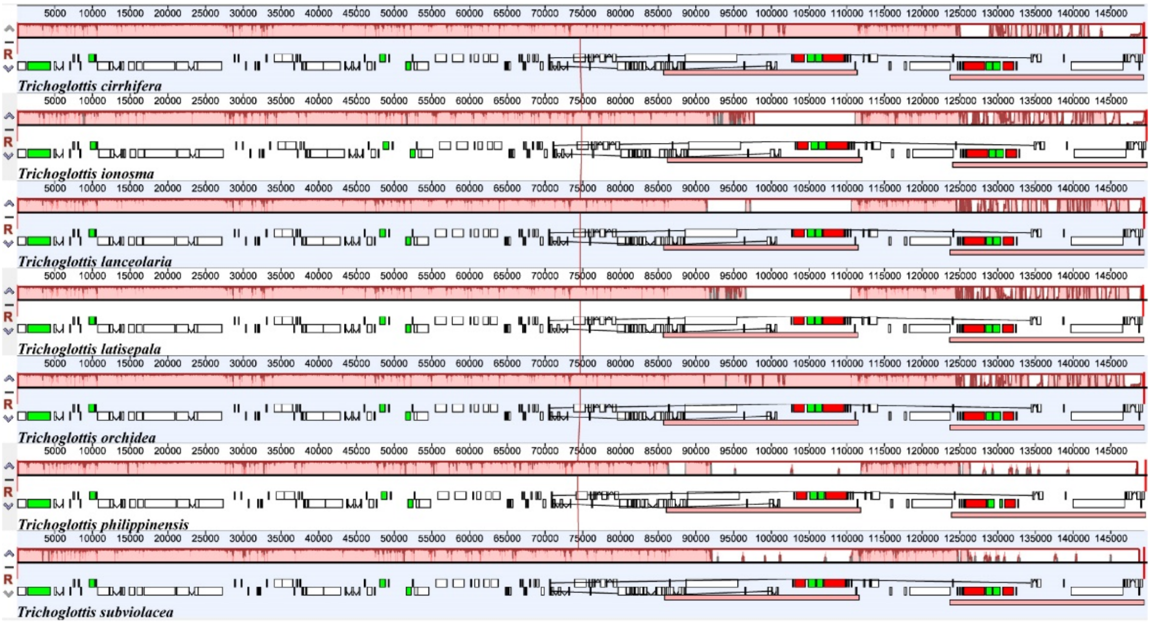

Supplementary Figure S1. Plastome comparison of seven species of *Trichoglottis* using a progressive MAUVE algorithm.

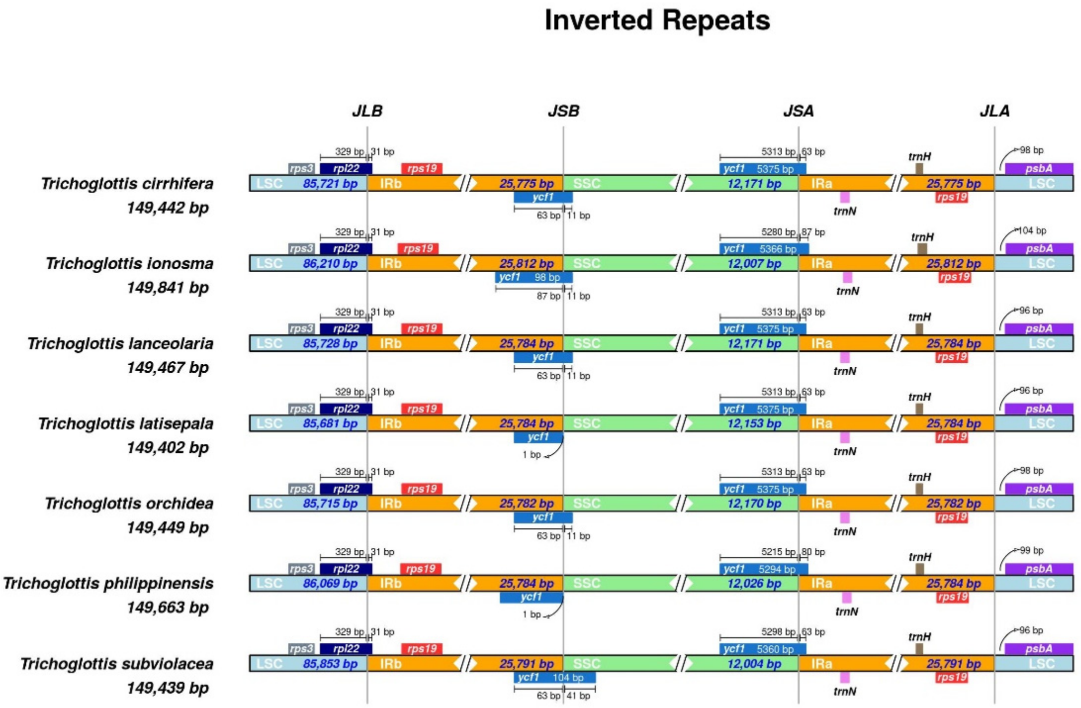

Supplementary Figure S2. Comparison of junctions between the LSC, SSC, and IR regions among seven *Trichoglottis* plastomes.

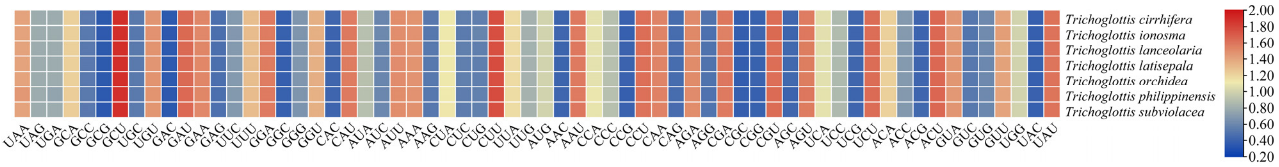

Supplementary Figure S3. The RSCU values of concatenated 68 protein-coding genes for seven plastomes. Color key: the red values mean higher RSCU values and the blue values mean lower RSCU values.

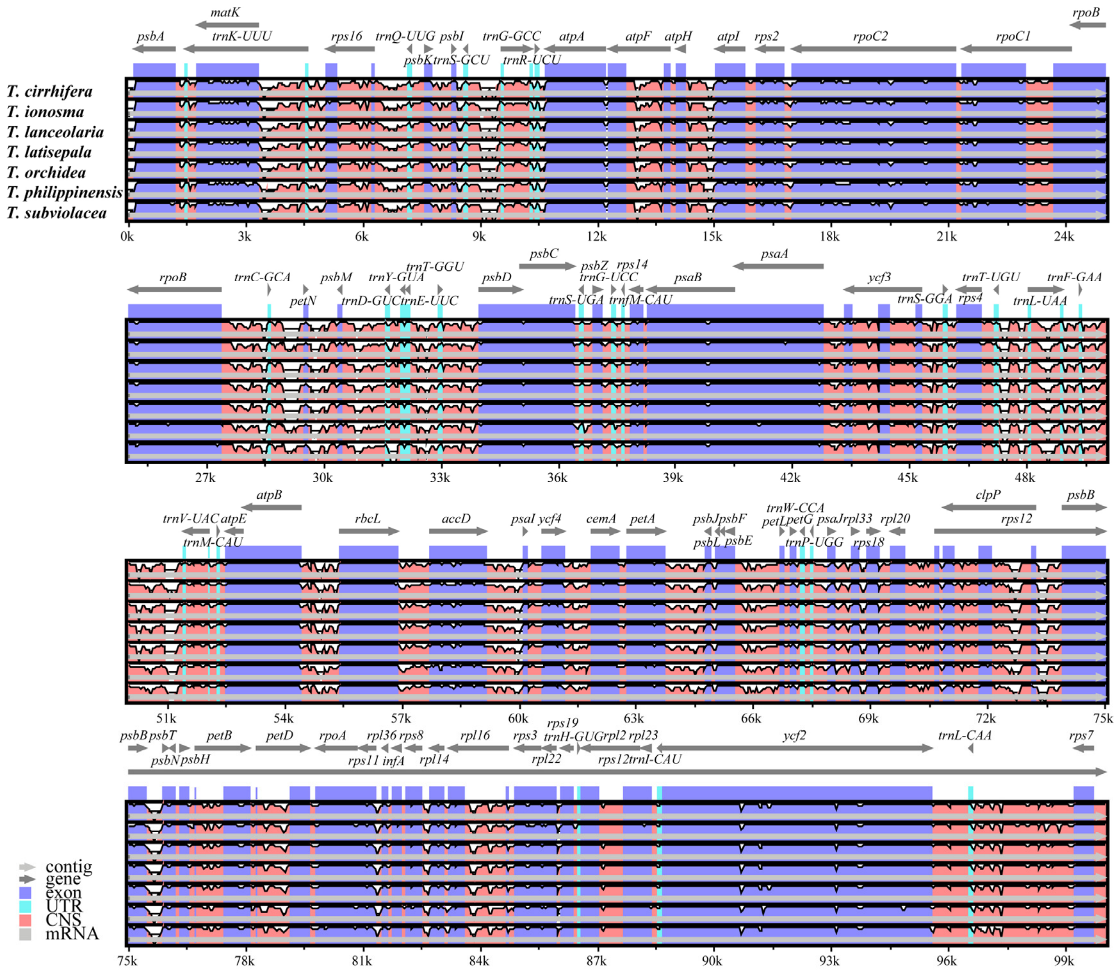

Supplementary Figure S4. Global alignment of seven *Trichoglottis* plastomes by mVISTA with *Thrixspermum centipeda* as reference. The y-axis shows the coordinates between the plastomes.

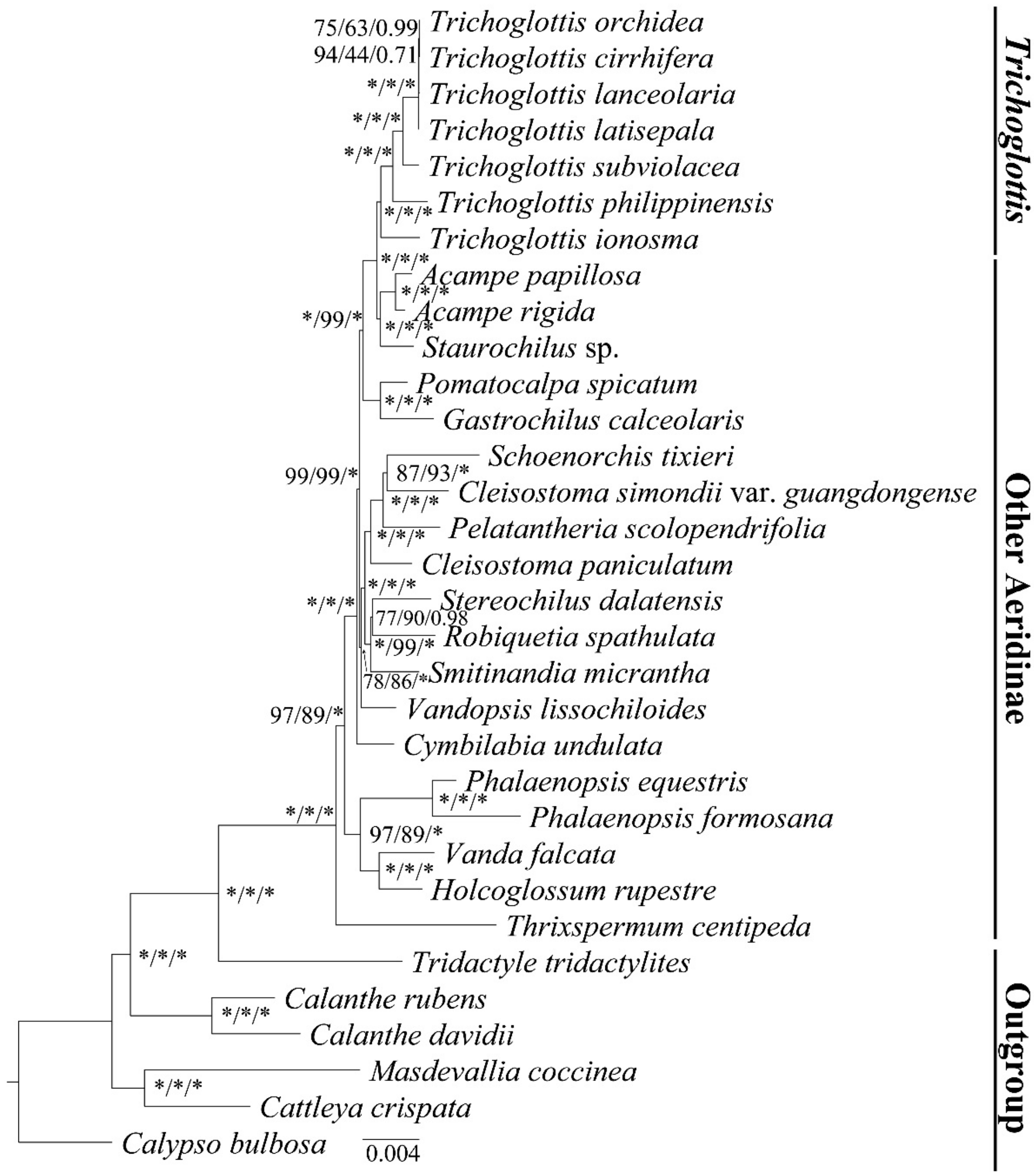

Supplementary Figure S5. Phylogenetic tree obtained by maximum-likelihood analysis based on 68 protein coding-genes. The numbers near the nodes are bootstrap percentages and Bayesian posterior probabilities (BP_{ML}, BP_{MP}, PP). A dash (-) indicates that a node is inconsistent between the topology of the MP/ML trees and the Bayesian tree; *node is 100 bootstrap percentage or 1.00 posterior probability

Supplementary Table S1. Source and voucher information for this study. Voucher specimens were deposited in the herbariums of Forestry College of Fujian Agriculture and Forestry University (FJFC) and National Center for Biotechnology Information (NCBI).

Supplementary Table S2. The details information of simple sequence repeats (SSRs).

Supplementary Table S3. The details information of long repeats.

Supplementary Table S4. The details information of relative synonymous codon usage (RSCU).

Supplementary Table S5. The nucleotide diversity of plastome and 68 protein coding genes in *Trichoglottis*.
